# Battered and Brain Injured: Assessing Knowledge of Traumatic Brain Injury Among Intimate Partner Violence Service Providers

**DOI:** 10.1089/jwh.2018.7299

**Published:** 2019-07-16

**Authors:** Halina (Lin) Haag, Sandra Sokoloff, Nneka MacGregor, Shirley Broekstra, Nora Cullen, Angela Colantonio

**Affiliations:** ^1^ Lyle S. Hallman Faculty of Social Work, Wilfrid Laurier University, Kitchener, Canada.; ^2^ Department of Occupational Science and Occupational Therapy, University of Toronto, Toronto, Canada.; ^3^ WomenatthecentrE, Toronto, Canada.; ^4^ West Park Healthcare Centre, Toronto, Canada.; ^5^ Rehabilitation Sciences Institute, University of Toronto, Toronto, Canada.; ^6^ Toronto Rehabilitation Institute—UHN, Toronto, Canada.

**Keywords:** traumatic brain injury, intimate partner violence, women, support interventions, domestic violence

## Abstract

***Background:*** Traumatic brain injury (TBI) as a result of intimate partner violence (IPV) is a significant health concern; yet, little is known about the intersection between the two. Existing research is scarce, limiting the ability of health care providers to develop effective supports. This pilot project surveyed the IPV support community in Toronto, Canada to understand the degree of existing TBI-specific knowledge and relevant services available among these service providers and to seek to bridge the divide between research and practice by developing a national knowledge-to-practice network to support brain-injured women survivors of IPV.

***Materials and Methods:*** In phase 1, 68 agencies providing IPV support services were invited to complete an anonymous online survey. In phase 2, 22 stakeholders attended a workshop held to disseminate existing knowledge, develop a national knowledge-to-practice network, and determine next steps in research and practice.

***Results:*** The results highlighted a general lack of TBI awareness and understanding among IPV service providers. In addition, participants stated that frontline workers and women survivors of IPV alike do not recognize signs or symptoms of TBI. Recommendations addressing research gaps, professional and public education, and service development were identified and are discussed herein.

***Conclusions:*** The identified lack of TBI knowledge among IPV service providers highlights the immediate need to increase education among management and frontline workers. Further investigation identifying best practices for knowledge transfer are suggested. The development of a national strategy addressing education, research, and funding is critical for successful uptake and integration of TBI-sensitive services within the IPV sector.

## Introduction

The World Health Organization (WHO) estimates 1 in 3 women are beaten by intimate partners over their lifetime.^1^ Also referred to as domestic or spousal violence, intimate partner violence (IPV) encompasses physical, sexual, and emotional abuse, and controlling behaviors. Globally, IPV affects women of all ages, socioeconomic groups, cultural backgrounds, and abilities.^2^ It is the primary cause of physical injury to Canadian women aged 15–44 years and is associated with high rates of mental illness, unemployment, and poverty.^3^ Most injuries are from battery to the face, head, and neck, including strangulation,^4^ leaving survivors vulnerable to traumatic brain injury (TBI). TBI is defined as “an alteration in brain function, or other evidence of brain pathology, caused by an external force.”^5^ It is important to note that while hypoxic-ischemic brain Injury, caused by oxygen deprivation from strangulation, is not usually classified as a traumatic brain injury, it does result in similar challenges postinjury^6,7^ and, as such, is included herein.

According to the WHO, “Traumatic brain injury is the leading cause of death and disability in children and young adults around the world.”^8^ It is associated with permanent cognitive, physical, psychological, and social dysfunction, including elevated rates of mental illness, and carries major personal and economic repercussions, such as increased medical costs and higher rates of unemployment, homelessness, and poverty.^9,10^ Repeated trauma to the head can result in symptoms such as fatigue, depression and mood changes, memory loss, confusion, aggression, and impaired judgment, and can lead to dementia and other chronic health conditions.^11–13^ Because of the high incidence of cranial assaults and the chronic nature of IPV, women survivors are at an increased risk for TBI with reported rates of 35–80%.^14^

Although this is an emerging topic in women's health, examination of this intersection and the implications for health care and service providers is limited. Despite widespread attention to sport and military-related concussion/TBI, WHO information on consequences of IPV, including injuries to the head, face, and neck, does not include brain injury.^2^ Whereas there are distinct bodies of literature examining both IPV and TBI, only a few studies directly address the correlation between them; a scoping review conducted by the authors (in press) identified 20 English language original research studies expressly considering the intersection.^15^ Existing studies have identified a relationship between IPV/TBI and a range of biopsychosocial challenges faced by survivors,^3,7,16–18^ and explored various health care contexts,^19–23^ as well as survivor categories such as veterans,^24^ ethnic groups,^25^ and marginalized women.^26^ Yet, although the identified studies focused on prevalence rates, identification and screening, and recommendations for health care professionals, none of them address the state of TBI knowledge among frontline workers, an area highlighted as being of key importance.^7^ Further investigation has been recognized as a priority both by researchers^3,14–16,18,27^ and in mainstream media.^28–31^

To address this gap, we conducted a pilot project surveying the IPV support community in Toronto, Canada to better understand the degree of existing TBI-specific knowledge and relevant services available, identify ways to improve survivor health and wellbeing, and bridge the divide between research and practice by developing a national knowledge-to-practice network to support brain injured women survivors of IPV. The objectives were to (1) explore service providers' knowledge of TBI and its impact on IPV survivors; (2) identify existing TBI-specific IPV services and the needs, barriers, and facilitating factors in TBI/IPV knowledge acquisition and translation; and (3) develop recommendations for tackling service and research gaps, and create a network of stakeholders to advance a national agenda addressing those gaps. A secondary goal was to provide education and training for survey participants who expressed an interest in learning more about TBI among their client populations.

## Materials and Methods

Meeting the first two research objectives, a 12-question survey (phase 1) was created by the research team. After consultation with survey design specialists at the University of Toronto and community-based partners with service provision experience and review of similar survey tools in other contexts, it was determined that the survey takes 10–15 min to complete, plain language should be used throughout, and questions should target knowledge of and familiarity with common TBI identifiers and appropriate care provision. A comprehensive list of 68 organizations within the Greater Toronto Area (GTA) that self-identified as offering support services to women survivors of IPV was compiled. Each organization received an email invitation to participate in the anonymous online survey, and two subsequent follow-up reminders were sent out during the next 2 months. Each organization was asked to have one representative respond.

To address our third objective and secondary goal, a workshop (phase 2) was held to disseminate knowledge, develop a national knowledge-to-practice network, and determine next steps in research and practice. Participants were recruited from phase 1 survey respondents, snowball sampling, and direct invitation of stakeholders beyond the GTA, drawing upon established contacts with service providers and women survivors of IPV. Participants included women survivors, frontline workers, administrators, and representatives from national and provincial disability and brain injury advocacy groups, provincial funding bodies, and national shelter organizations. The workshop was held in Toronto, Ontario immediately before the Brain Injury Canada Annual Conference.

The workshop consisted of an information session on IPV-related TBI, three small breakout discussion groups and a subsequent full group discussion to share findings, and a public panel. Based on participatory models of qualitative research methods, discussion questions were designed to elicit in-depth information about existing knowledge and service gaps and gather recommendations for next steps in research, practice, and knowledge dissemination. Breakout groups included at least one representative from each participant category listed above to achieve triangulation of the data, and an experienced facilitator and a note-taker from the research team so that at least two members were present. Session summaries were presented to the collective to identify key themes and prioritize next steps. Instead of the research team determining emergent themes postevent through an inductive process, themes were identified collaboratively by the team and participants at the end of the workshop. To be identified as a theme and included here, members of each breakout group had to be in agreement, it had to emerge across all three breakout groups, and the collective had to agree upon it being of particular significance. Following standard qualitative methodology, no specific quantitative threshold was required for inclusion. Discussions were recorded by members of the research team in written and audio formats. This information was then collated and summarized by at least three members of the research team. Ethical approval was sought and granted by Ethical Review Boards of both the University of Toronto and the Toronto Rehabilitation Institute—UHN. All participants provided written informed consent to participate in the study and for publication. This article reports on the results of both phases of the project.

## Results

The response rate was 28% with 19 organizations submitting surveys primarily completed by frontline workers and management staff. The majority offered a wide variety of IPV support services including shelter, professional and/or peer counseling, substance use interventions, legal and medical support, education, referral, and settlement support and had annual budgets exceeding 1 million dollars (Table 1). Twenty-two participants attended the workshop, including women survivors of IPV and representatives of local (GTA), provincial, and national organizations providing both IPV and TBI services. The following results include both phases of the project and are presented by research objective.

**Table 1.** Estimated Annual Budget for Intimate Partner Violence Support Organizations

*Estimated annual budget (CDN $)*	*Rate of response, %*
<$250,000	16.6
$251,000–$499,999	16.6
$500,000–$1,000,000	16.6
>$1,000,000	50

### Objective 1: explore service providers' current knowledge of TBI and its impact on IPV survivors

The results highlighted a general lack of awareness and understanding of TBI among IPV support service providers. *Survey:* The majority (84%) of survey respondents reported no previous TBI training or education relevant to their work with women survivors of IPV. Although it is possible respondents reported training as not relevant as opposed to nonexistent, qualitative investigation reported hereunder supports our interpretation of the data. Respondents who had received some training reported formal in-house information sessions provided by a national brain injury organization with a small component of various training sessions. Only 5% of respondents thought that between 51% and 80% of their clients could have a TBI (Table 2). This lack of awareness was also evident in participants' reported comfort level in recognizing signs and symptoms of TBI: all survey respondents reported feeling totally unprepared to only somewhat prepared to identify signs or symptoms of TBI among their clients (Table 3). Forty-two percent of respondents ask clients about concussion, 47% ask about blows to the head, face, or neck, 63% stated they ask clients about loss of consciousness, and 68% enquire about strangulation.

**Table 2.** Expected Rate of Traumatic Brain Injury in Intimate Partner Violence Client Population

*Expected rate of TBI in IPV survivors, %*	*Rate of response, %*
<10	29
Between 11–25	14
Between 26–50	57
Between 51–80	0
>80	0

TBI, traumatic brain injury; IPV, intimate partner violence.

**Table 3.** Rate of Preparedness to Identify Traumatic Brain Injury Signs and Symptoms in Clients

*Rate of preparedness*	*Rate of response, %*
Totally prepared	0
Mostly prepared	0
Somewhat prepared	17
Mostly unprepared	50
Totally unprepared	33

*Workshop:* This gap in knowledge was further supported by workshop participants who also reported little or no previous TBI education or training. Across each discussion group, participants stated that frontline workers and women survivors of IPV alike do not recognize signs or symptoms of TBI. Participants did not connect information about sports-related TBI made familiar through media coverage with injuries sustained through IPV. Although service providers often asked women about concussion, strangulation, or hits to the head, face, and neck, and referred them to medical services, they indicated they were unaware of other support services, and expressed concern that identifying and labeling their clientele as brain injured could have negative legal implications for their clients. Participants were of the opinion that women's shelters were short-staffed and that there is limited access to TBI-related services in smaller or rural communities. The following points were made in the discussion groups:

- Service providers were unprepared to identify signs and symptoms of TBI because they did not know enough about it, acknowledging this prevents them knowing what to ask and when.
- Widespread lack of awareness of the IPV/TBI connection and/or cognitive and behavioral outcomes prevents survivors from self-identifying and workers from asking appropriate questions, potentially resulting in large numbers of hidden TBI within client populations.
- Lack of knowledge about available resources results in referrals into medical systems rather than brain injury support systems that can be problematic as financial support in the health care system runs out in short time.
- A perception that health care for TBI patients is underserviced.
- Limitations for shelters in terms of staffing numbers; adding more information that needs to be delivered means more staffing required.

Support personnel often pressure survivors to conform to standards of reliability and credibility expected within contexts such as court systems and child protection, ostensibly for the client's own benefit, not realizing that an inability to do so may be indicative of functional deficits from an undiagnosed brain injury rather than behavioral issues. This has implications for parenting as daily challenges faced by women with TBI may have a significant impact on their ability to navigate legal systems and child custody arenas, as noted by participants.

### Objective 2: identify TBI-specific services being provided within the IPV survivor support community, and the needs, barriers, and facilitating factors in TBI/IPV knowledge acquisition and translation

The impact of the identified knowledge gap on existing services became evident when survey respondents were asked about current practices. *Survey:* Despite 100% of respondents' reporting that they routinely provide information and referral services to women survivors of IPV, fewer than half provided TBI-relevant information (Table 4), and most respondents reported sending clients to medical professionals, such as family physicians, hospital emergency centers, and walk-in or urgent care clinics (Table 5).

**Table 4.** Relevant Information Provided to Clients When Traumatic Brain Injury Signs or Symptoms Present

*Visible or described sign or symptom of TBI*	*Yes (%)*	*No (%)*	*N/A (%)*
Hits to head, face, neck (with fist or object)	33	50	17
Black eyes	33	67	0
Being strangled or “choked”	33	50	17
Being smothered	17	67	16
Being thrown against wall or floor	17	83	0
Being pushed down stairs	17	83	0
Being shaken	0	83	17
Knocked out teeth	33	67	0
Loss of consciousness	33	67	0
Loss of memory or trouble remembering new things	33	67	0
Headache	33	67	0
Difficulty concentrating	33	67	0
Ringing in the ears	17	83	0

**Table 5.** Rate of Referral to Outside Agency for Traumatic Brain Injury-Specific Services

*Referral for TBI-specific supports by agency type*	*Yes (%)*	*No (%)*	*N/A (%)*
Walk-in clinic	66	17	17
Emergency room	72	14	14
Urgent care clinic	50	33	17
Family physician	67	17	16
Sexual Assault/Domestic Violence Care Centre	50	33	17
Legal assistance	50	33	17
Brain injury support organizations	17	50	33
Counseling	85	15	0
Disability support services	50	50	0

*Workshop:* Participants noted numerous barriers to providing effective support services. Participants identified funding as a significant hurdle:

- Funding bodies are complex and smaller shelter organizations lack skills in gaining access to funds.
- Silo effect in funding across federal and provincial funders: complexity of IPV and different funding policies across provinces result in an inconsistent national approach to addressing violence against women.
- Funders are perceived to be unaware of the intersection of IPV and TBI; need for education among senior decision makers.

Workshop participants who work with survivors of IPV expressed concern over frightening, overwhelming, or stigmatizing their clients, and specter of stigma was considered another service barrier.

- Stigma and shame associated with IPV/TBI might prevent women from self-identifying and seeking appropriate help, or might influence legal professionals' decisions about parenting skills and suitability.
- Social pressure to remain silent: risk of repeated violence if you speak out, fear/risk of losing children, feelings of guilt, and religious beliefs about women's social roles.

Workshop participants discussed the use of TBI screening tools during the intake process. They felt that using a screening tool could be beneficial for providing appropriate services and referrals, but could also lead to stigmatization. However, without a diagnosis, access to health care, rehabilitation, and legal supports may be severely limited. Participants pointed out that screening in smaller communities that do not offer TBI services could raise expectation and hope that cannot be met by available health care options.

All participants identified education among stakeholders and public awareness as key to supporting women survivors through early intervention and prevention. They agreed that educating women survivors can be reassuring and self-affirming if done skillfully with a goal to normalize TBI by simplifying/de-medicalizing the language and allowing survivors to make connections to their own experiences. Frontline workers and other staff, decision makers, and workers across legal and health care systems, including first responders, funding organizations, journalists, police and correctional services, and violence against women and brain injury advocacy groups were listed as target audiences for awareness and education. Mandatory training in postsecondary professional programs, education in secondary schools, public awareness campaigns, and public service announcements were identified as potential facilitators to TBI/IPV knowledge acquisition and translation.

### Objective 3: develop strategies and recommendations for next steps in addressing service and research gaps, and develop a network of stakeholders to advance a national agenda addressing these gaps

*Survey:* Eighty-eight percent reported they were both willing and able to create TBI inclusive services if proper training and support are available. Respondents indicated a strong commitment to providing the best client services possible and reducing barriers to access and uptake.

*Workshop:* Participants unanimously agreed that education and training is an immediate and essential strategy for addressing gaps across the spectrum of services, and that collaboration among stakeholders is crucial for successful service provision and knowledge transfer. Community-based peer support, a unified service hub, and use of a feminist, trauma-informed model of care among all service providers were suggested approaches to developing an effective response. Although immediate and local solutions were sought, participants strongly agreed that a national plan addressing systemic and political barriers along with multiple stakeholders' needs, backed up by research evidence, is needed. The following research priorities were identified:

- Identifying Canadian epidemiological data, particularly prevalence rates, and longitudinal studies with large data sets that can be used to influence policy, funding, and programming decisions.
- Obtain qualitative, experiential data gathered from women survivors to add depth and richness to quantitative data and inform about survivor impact.
- Determine best practices for knowledge transfer to women survivors of IPV.
- Identify a TBI screening tool that is sensitive to the IPV context.
- Explore the implications of screening for and diagnosis of women with TBI related to IPV to determine if it is harmful or helpful.
- Determine a possible relationship between the pathophysiology of IPV, TBI, and Alzheimer's disease.

Participants collectively agreed that research initiatives be collaborative and interdisciplinary in design; that they must use an intersectional and anti-oppression lens; and that in all stages of the process they must include Indigenous populations, criminalized women, women of ethnic backgrounds, and sex workers.

## Discussion

Significant implications for practice and research can be drawn from these results. First and foremost is the suggested need for a widespread education campaign addressing the current lack of understanding about the relationship between TBI and IPV among the IPV service sector, health and legal professions, and the general public. This is consistent with WHO recommendations for the prevention of and response to IPV against women globally, which call for extensive campaigns to increase understanding among health services providers and other support sectors, along with previous reviews that note the need for broader education within health care professionals regarding the intersection of IPV and TBI.^1,7,17^ As highlighted by Campbell et al.,^7^ priority focus on frontline workers and support agency staff to enable earlier identification of possible TBI among clients and the adaptation of existing services to better support women survivors is critical. In addition, training and support in best practices for identification of women with TBI and intervention within an IPV context will need to be developed and implemented. It is also important that information on TBI become readily available for people involved in relevant legal professions such as family courts, domestic violence courts, and criminal courts, along with individuals working in child protection arenas. TBI and its subsequent sequelae may well have an impact on survivors' abilities to successfully navigate these systems.

Second, there is a substantial need for extensive research in this field, particularly in the following areas: identification and screening, best practices for TBI-sensitive intervention and support including shelter design and environment, rehabilitation strategies for return to work and parenting, barriers to successful navigation of current legal and child protection systems, and long-term implications for health such as the development of progressive degenerative neurological diseases. This is again in line with current WHO practices to increase the evidence base on IPV demographics, occurrence rates, and consequences worldwide.^1^ Empirical evidence is needed to support sustainable funding, influence relevant policy decisions, redirect health care initiatives, and develop educational tools. Although there is limited research in this field, our call for more exploration echoes those made by previous authors.^3,7,13–15,17,27^ Furthermore, our results suggested that ideally, research teams should comprise interdisciplinary researchers, IPV survivors, and support professionals, and should strive to include quantitative and qualitative inquiry to provide a rich understanding.

Finally, the study highlighted the need for a national strategy addressing this issue. The development of an interconnected network of researchers, IPV service providers, women survivors, legal professionals, and policy decision makers is a high priority. The WHO has specifically identified the need for a multisectoral approach to the issue of IPV in general and the results of this pilot study reiterate this need.^1^ This study provided an opportunity for first steps to be taken in this endeavor, but consistent follow-up on a national scale is necessary. Relationships across disciplines and professions must be fostered to address barriers to knowledge translation and uptake. To be successful, it is essential that this network engage in simultaneous efforts to increase public awareness and professional knowledge, develop new policy directions, and seek sustainable program funding to support women survivors.

## Limitations

Although the rate of return for the survey was reasonable, the small sample size limits generalizability. It is also possible that organizations offering relevant services were overlooked. The funding program for this pilot project is designed to support research at a local level, but limiting our geographical boundary to the GTA for the survey could have resulted in representation of organizations with considerable financial and community resources that might not be available in other areas. Similar small-scale projects in other communities across the country, or a large-scale national project are needed.

## Conclusions

Women exposed to IPV are at an increased risk of TBI. Despite this, little information exists exploring the implications for women survivors or support service providers. This novel, multifaceted project assesses TBI knowledge among service providers, identifies gaps in service provision and knowledge transfer, and generates recommendations to address those gaps. The identified lack of knowledge highlights the need for immediate attention to increase education among both management and frontline workers. Further investigation to identify best practices for training and education is suggested. The development of a national strategy addressing education, research, and funding is critical for successful uptake and integration of TBI-sensitive services within the IPV sector; a sustainable knowledge-to-practice network with potential allies is recommended to better enable implementation. Finally, further research into prevalence, support, and prevention is required to develop effective interventions and support much needed changes in funding and policy. Research should rely on a participatory and intersectional model that privileges the voice of survivors, using both quantitative and qualitative methods to provide a comprehensive picture.

## Availability of Data and Materials

Although data generated or analyzed during the study cannot be shared publicly for reasons of confidentiality, it may be made available from the corresponding author upon reasonable request.
